# Effectiveness of ultrasound guided interfascial hydrodissection with the use of saline anesthetic solution for myofascial pain syndrome of the upper trapezius: a single blind randomized controlled trial

**DOI:** 10.3389/fresc.2023.1281813

**Published:** 2023-12-11

**Authors:** Charidy Suarez-Ramos, Consuelo Gonzalez-Suarez, Ivan Neil Gomez, Maria Katherine Gonzalez, Philippe Hubert Co, Jose Alfonso Llamas

**Affiliations:** ^1^ Physical Medicine and Rehabilitation, Our Lady of Lourdes Hospital, Manila, Philippines; ^2^ Research Center for Health Science, University of Santo Tomas, Manila, Philippines; ^3^ Department of Occupational Therapy, College of Rehabilitation Science, University of Santo Tomas, Manila, Philippines; ^4^ Center of Health Research and Movement Sciences, University of Santo Tomas, Manila, Philippines; ^5^ Physical Therapy and Rehabilitation Medicine Department, Gat Andres Bonifacio Medical Center, Manila, Philippines; ^6^ Health Hub Physical Therapy, Rehabilitation Medicine and Orthopedics Clinic Inc., Manila, Philippines; ^7^ Physical Therapy and Rehabilitation Medicine Department, Region I Medical Center, Dagupan City, Pangasinan, Philippines

**Keywords:** myofascial pain syndrome, interfascial hydrodissection, upper trapezius, upper back pain, wet needling

## Abstract

**Background:**

Myofascial pain syndrome (MPS) is described as pain that arise from myofascial trigger points (MTrPs) which is a hyperirritable spot within a taut band of skeletal muscle. A newer needling technique called the interfascial hydrodissection (IH), wherein anesthetic saline solution (ASS) is injected between the fascia of the muscles using ultrasound as guide. It is theorized that this technique blocks the nerve branches and improve gliding in between the muscle and fascia.

**Objective:**

To determine the short and long-term effects of interfascial hydrodissection using 2% Lidocaine and saline solution compared to dry needling with MPS of the upper trapezius on pain and quality of life using.

**Methods:**

This study is a single-blind randomized controlled trial where ultrasound guided IH with ASS was compared to dry needling (DN) of the MTrPs. Both groups were taught self-stretch exercises (SSE) to be done everyday after the procedure. Outcome measures were pain using the visual analogue scale (VAS) and quality of life assessment with EQ-5D-5l questionnaire. All participants were assessed by a blinded assessor before the intervention, immediately after, 10 and 30 min, one week, two weeks, four weeks, three months, and six months after the procedure. Data Analysis: Two-way mixed ANOVA and follow-up independent *T*-test were conducted for the outcome measures across several time points between the 2 groups.

**Results:**

A total of 46 participants with two dropouts were all included during the final analysis. Both groups demonstrated significant differences in VAS scores between baseline and the different time points, the IH + SSE group demonstrated the more significant effect size at as compared to the DN + SSE group. For EQ-5D-5l, no statistical differences were seen in all dimensions but there was a larger effect size for usual activities, pain/discomfort and anxiety/depression.

**Conclusion:**

Interfascial hydrodissection is a technique that can manage both short and long term symptoms of MPS. This could be utilized as an alternative management for those with chronic MPS of the upper trapezius.

**Philippine Health Research Registry ID:**

PHRR221003-005034.

## Introduction

Myofascial pain syndrome (MPS) is one of the most common causes of chronic musculoskeletal pain and is described by the presence of myofascial trigger points (MTrPs). MTrP is “a hyperirritable spot within a taut band of skeletal muscle that is painful on compression, stretch, overload, or contraction of the tissue, which usually responds with a referred pain that is perceived distant from the spot” (1). MPS affects up to 85% of the general population (2, 3). The pathophysiology of MPS is still poorly understood. Several theories to elucidate the origin of pain include central and peripheral sensitization, the integrated trigger point hypothesis, and the Cinderella hypothesis (4).

Two kinds of fascia are anatomically seen at the upper back which are the superficial and deep fascia. In myofascial pain syndrome, the deep fascia, which is a multi-layer fibrous sheath connecting the muscles and tendons via myofascial expansions, is more affected (5). Increase of hyaluronic acid chains within the layers of the deep fascia can lead to excessive stiffness causing intrafascial gliding impairment (6). These fascial structures are vastly innervated by free nerve endings which are also pain generators (7).

The treatment of MPS includes pharmacologic and nonpharmacologic means. Pharmacologic agents include acetaminophen, nonsteroidal anti-inflammatory drugs, benzodiazepines, tropisetron, lidocaine, and anticonvulsants. However, a review showed limited evidence of the efficacy of the drugs mentioned above (8).

Non-pharmacologic treatment includes physical therapy, manual therapy, and needling. Needling of the MTrPs, either by dry or wet type, is the mainstay of the interventional treatment. Dry needling is done by placing a needle into MTrPs using an in-and-out technique in different directions to deactivate the MTrPs, leading to muscle relaxation. Injectates such as lidocaine and botulinum toxin of MTrPs are used, which constitute wet needling (9). More recent systematic reviews and meta-analyses (9, 10) concluded that dry needling is effective vs. sham treatment. However, the two reviews have conflicting results in evidence when dry needling was compared to wet needling.

Recently, a newer technique in treating MPS is the interfascial block wherein anesthetic is injected between the fascia of the muscles using ultrasound as a guide. The fascia promotes movement by allowing one muscle or fiber to move independently and creating an interfascial space between muscles (11). Furthermore, it is postulated that muscle relaxation is achieved by blocking the sensitivity of the nerve endings embedded within the fascia. However, there are limited studies that determined its effectiveness in the treatment of MPS (12–14).

In this study, the researchers aim to determine the short and long-term effects of interfascial hydrodissection using 2% Lidocaine and saline solution compared to dry needling with MPS of the upper trapezius on pain using the visual analog scale (VAS) and quality of life using EQ-5D-5l questionnaire. It also reported on the possible adverse events of the two treatments.

## Materials and methods

This study was approved by the Research Ethics Committee of the University of Santo Tomas Hospital with protocol reference no. REC-2021-01-006-TR-A1/CR.

Type of Study: This single-blind randomized controlled trial includes all patients who complain of upper back pain and are newly diagnosed with MPS.

### Participants

#### Sample size calculation

The sample size was computed using G*Power ver. 3.1.9.4 using a prior analysis for a repeated ANOVA (within-between interaction) at a postulated moderate effect size (*f* = 0.25), power of 0.80 at *α*= 0.05. Based on the sample size analysis, the required sample is *n* = 20 per group. Accounting for possible dropouts, a 20% inflation rate was adopted. Thus, this study recruited 46 subjects divided between the two groups.

Participants were between 20 and 50 years of age and were either male or female. They complained of upper back pain and met the clinical diagnostic criteria of Gerwin (15). The criteria include the presence of a taut band within the muscle, exquisite tenderness of the point of the taut band, reproduction of the patient's pain, local twitch response, referred pain, weakness, and restricted range of motion. The first three features are essential in the diagnosis of MPS. Exclusion criteria included pregnancy, cardiovascular or respiratory diseases, allergies, fibromyalgia, neurological disorders, renal or hepatic disorders, bleeding disorders, previous shoulder or neck surgeries, fear of needles, and patients currently having physical therapy and medication for neck pain or those with less than a month of interval from any intervention done.

#### Group allocation

Before participation, the subjects were oriented on the study objectives, possible outcomes, risks, and compensation. All participants signed the consent form.

Participants were assessed if they met the inclusion and exclusion criteria. They were randomly allocated into Group 1, interfascial hydrodissection with self-stretch exercise (IH + SSE) group, or Group 2, dry needling with self-stretch exercise (DN + SSE) group using EXCEL random generation of numbers. After which, the subjects underwent the assigned intervention.

### Intervention

#### Self-stretching exercises

All participants were taught self-stretching exercises and were given an instructional guide with a return demonstration on muscle stretching exercises. The exercises were performed daily. The researcher sent weekly text messages reminding them to do the exercises. The participants were also advised not to undergo any other interventions for MPS during the research period.

#### Interfascial hydrodissection

The intervention was performed by a registered musculoskeletal sonologist who has been performing musculoskeletal ultrasounds for more than ten years. Before the intervention, the sonologist palpated the tender nodule to locate the injection site. An ultrasound machine (GE Logic Q) with a linear transducer (5–13 MHz) was used to visualize the fascia during the procedure. Participants were seated with their necks flexed. Topical anesthetic using lidocaine 2% prilocaine 2% (EMLA) cream with transparent film dressing (Tegaderm) on the target site and ice compress was applied for 30 min. Aseptic preparation of the needle insertion site was performed with Propanol Benzalkonium Chloride and sterile drapes. With the use of in plane approach, the sonologist injected the saline anesthetic solution (1 cc 2% lidocaine + 5 cc NSS) between the fascia of the trapezius muscle and the fascia of the muscle directly under it with ultrasound guidance. This was followed by applying direct pressure and dressing over the injection site. Figure 2 shows the pre and post procedure scan of the upper trapezius that underwent hydrodissection.

#### Dry needling

A physiatrist performed the intervention who was trained to perform dry needling. The patient was seated in a relaxed position with the muscle to be treated exposed. Propanol Benzalkonium Chloride was applied as an antiseptic. The physiatrist identified the trigger point by palpating for taut bands. The topical anesthetic was applied using lidocaine 2% prilocaine 2% (EMLA) cream with transparent film dressing (Tegaderm) on the target site. A pincer grip technique was employed to lift the skin gently. A high-quality, sterile, disposable, solid filament needle (gauge 0.25 mm by 40 mm length) was inserted directly through the skin. The depth of needle penetration was sufficient to engage the MTrP. Dynamic needling which is an up and down pistoning motion in and out of the muscle was performed. After which, the needle was withdrawn, and pressure was applied (16).

#### Outcome measures

VAS scores and adverse events were obtained prior to the procedure, immediately after the procedure, 10 min and 30 min after the procedure. For one week, two weeks, one month, three months, and six months follow-up, the VAS scores, adverse effects, and EQ-5D-5l scores were recorded. They were contacted via phone to obtain the information for the follow up. All data prior and after the procedure were taken by an assessor blinded to the intervention they were allocated.

Demographic data: age, gender, and nature of work.

Pain assessment: visual analog scale (VAS) with scores ranging from 0 to 10.

EQ-5D-5l: EQ-5D-5l is a health utility instrument with 5 dimensions namely mobility, self-care, usual activities, pain/discomfort and anxiety/depression are assessed with scores ranging from 1 to 5 per dimension. This questionnaire has an excellent reliability with most psychometric studies having an interclass correlation (ICC) of 0.75–1.00 and moderate to strong validity of the dimensions and levels used. The Filipino or English version was administered based on the participant's preference (17).

Adverse events: Acute bleeding right after the procedure if any were noted. Any pain felt in the upper back, neck, head and upper extremities were observed. Limitation of motion of the cervical area and both upper extremities as well as numbness and weakness of the same areas mention were asked to be observed by the participants.

### Data analysis

All data were entered in a purpose-built EXCEL file. SPSS package (version 25) was used. Mean, standard deviations, and percentages were employed for the descriptive data, VAS score, adverse events and results of EQ-5D-5l across several time points. Two-way mixed ANOVA and follow-up independent *T*-test were conducted for the outcome measures across several time points between the IH + SSE and DN + SSE groups. Intention to treat analysis was used. The estimated effect size was reported using partial ETA-squared and Cohen's d, and precision using a 95% confidence interval. A *p*-value of less than 0.05 was considered significant.

## Results

### Participants

**Figure 1:**
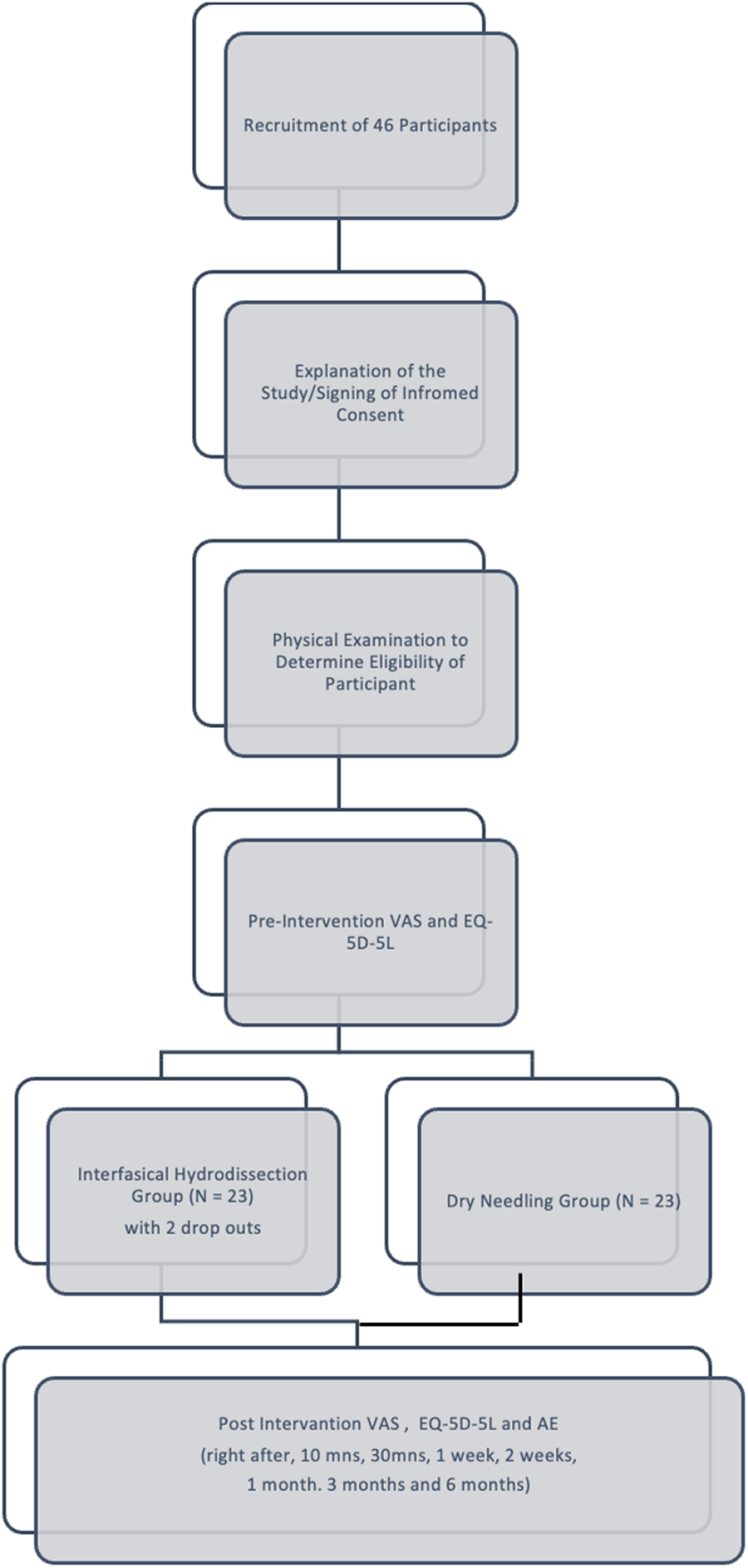
Consort diagram.

Forty-six participants were screened were randomly assigned to either the IH + SSE (*n* = 23) and DN + SSE (*n* = 23) group. The study has two dropouts, one was due to a vehicular accident, and another was an undiagnosed cervical radiculopathy, which was validated by nerve conduction and electromyography studies. All participants were included in the final analysis (Figure 1).

The mean age of the participants was 31.70 ± 5.70 years, mostly females (*n* = 31, 67.4%). There was no significant difference between the age and gender of the two groups. There was no significant difference in the presenting symptoms of the participants in each trial arm except for local twitch, which was present significantly in the II-SSE group (23 vs. 14, *p*-value = 0.000). The most common signs of MPS were taut band, tenderness, pain, and local twitch. All participants have been experiencing chronic pain. The pre-treatment pain score and the EQ-5D-5l results were also not statistically different for both groups (Table 1).

**Table 1:** Participant characteristics.

Participant characteristics	Hydrodissection group	Dry needling group	Total	*p*-value
Demographics				
*n*	23	23	46	
Age (years; Mean ± SD)	31.09 ± 5.87	32.30 ± 5.60	31.70 ± 5.70	0.475
Gender (*n*,%)	Female: 17, 73.9%	Female: 14, 60.9%	Female: 31, 67.4%	0.356
MPS symptoms				
Taut band	Present: 23, 100%	Present: 23, 100%	Present: 46, 100%	
Tenderness	Present: 23, 100%	Present: 22, 95.7%	Present: 45, 97.8%	0.323
Pain	Present: 23, 100%	Present: 22, 95.7%	Present: 45, 97.8%	0.323
Local twitch	Present: 23, 100%	Present: 14, 60.9%	Present: 14, 30.4%	0.000
Referred pain	Present: 15, 65.2%	Present: 18, 78.3%	Present: 33, 71.7%	0.337
Weakness	Present: 3, 13%	Present: 2, 8.7%	Present: 5, 10.9%	0.645
Limitation of movement	Present: 19, 82.6%	Present: 16, 69.6%	Present: 35, 76.1%	0.310
Autonomic signed	Present: 23, 100%	Present: 23, 100%	Present: 46, 100%	
Outcome measures (Mean ± SD)				
VAS score	4.78 ± 1.78	5.04 ± 1.72		0.616
Mobility	1.30 ± 0.63	1.09 ± 0.29		0.142
Self-care	1.26 ± 0.54	0.26 ± 0.62		1.000
Usual activity	2.52 ± 0.79	2.26 ± 0.81		0.275
Pain/discomfort	2.61 ± 0.84	2.78 ± 0.74		0.459
Anxiety/depression	1.70 ± 0.88	1.61 ± 0.89		0.740

### Pain

The IH + SSE group had a significantly lower VAS score than the DN + SSE group immediately, 10 min, and 30 min after intervention. VAS scores after one week up to 6 months were not statistically different for both groups (Figure 3). The results of the Mixed two-way ANOVA showed that there was a significant main effect of time on VAS scores overall [*F*(8,352) = 39.00, *p* < 0.000, *η_p_*^2^ = 0.47, *β* = 1.00]. There was a significant interaction between time and grouping in terms of VAS scores [*F*(8,352) = 6.20, *p* < 0.000, *η_p_*^2^ = 0.12, *β* = 1.00]. The main effect of the intervention on the VAS scores of the participants was to found approaching statistical significance [*F*(1,44) = 3.80, *p* = 0.058, *η_p_*^2^ = 0.08, *β* = 0.48].

**Figure 2:**
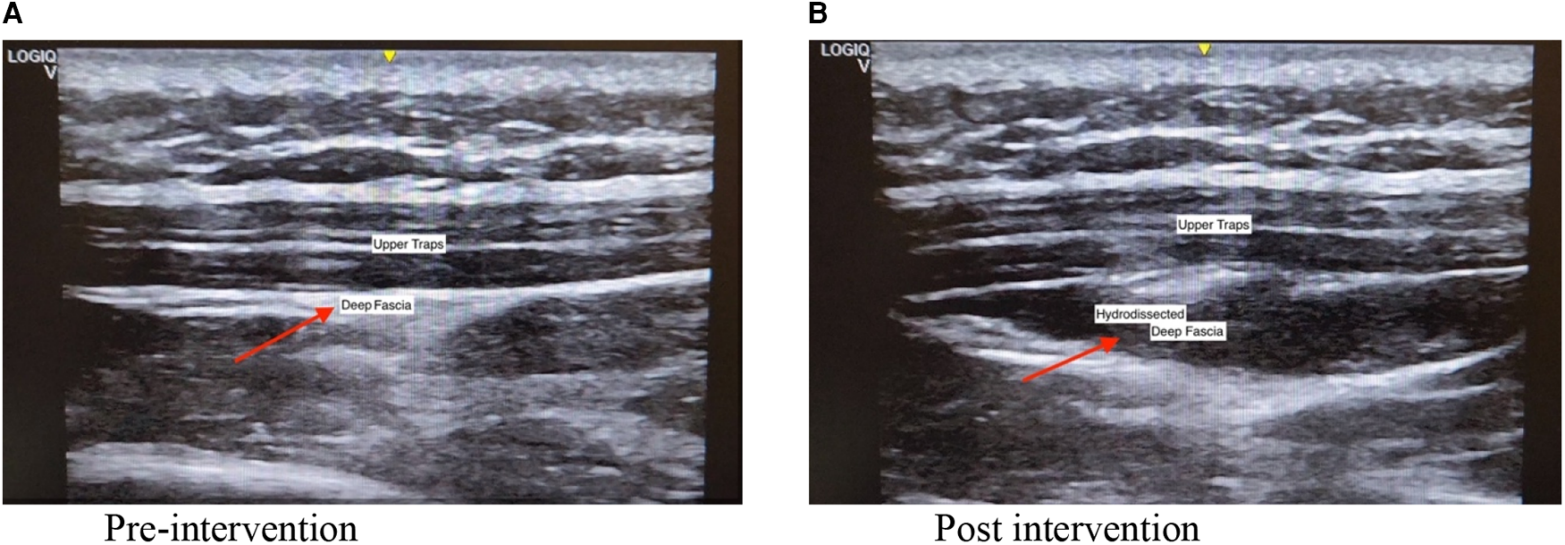
Ultrasound guided hydrodissection (**A**) pre-intervention (**B**) post intervention.

**Figure 3:**
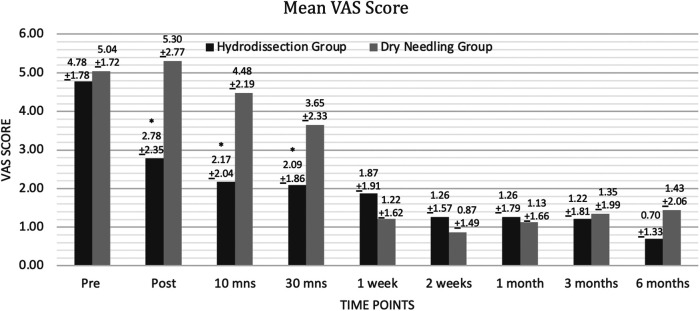
Mean VAS score.

Analysis was performed by looking at the difference in the pain scores using VAS at baseline and across the different post-intervention time points between the two groups. While both groups demonstrated significant differences in VAS scores between baseline and the different time points, the IH + SSE group demonstrated the more significant effect size at the following time points: immediately after, after 10 min, after 30 min, after three months, and after six months. The DN + SSE group had a higher effect size at one, two, and four weeks after intervention. Overall, the magnitude of intervention effects of IH + SSE may be most effective immediately after interventions and in the long term when compared to dry-needling (Figure 3).

### Mobility

No statistically significant difference was present with the mean scores and changes from baseline to different time points for both groups.

### Self-care

No statistically significant difference in self-care was noted for both groups across the different time points. Statistically significant mean changes in self-care were seen in the IH + SSE group from baseline and two weeks up to 3 months post-intervention but not for the DN + SSE group (Table 3).

**Table 2:** Summary of paired samples *t*-tests for pain scores between groups.

Timepoint comparison of pain scores	Hydrodissection group (*n* = 23)			Dry-needling group (*n* = 23)		
	M ± SD	*p*-value	Cohen's *d*	M ± SD	*p*-value	Cohen's *d*
Before—After	2.00 ± 2.00	0.000	2.05	−0.26 ± 3.06	0.687	−0.17
Before—10 months post	2.61 ± 1.85	0.000	2.88	0.57 ± 2.56	0.300	0.45
Before—30 months post	2.70 ± 1.92	0.000	2.88	1.39 ± 2.64	0.019	1.08
Before—1 week post	2.91 ± 2.64	0.000	2.25	3.83 ± 2.62	0.000	2.98
Before—2 weeks post	3.52 ± 2.23	0.000	2.25	4.17 ± 2.42	0.000	3.52
Before—4 weeks post	3.52 ± 2.23	0.000	3.22	3.91 ± 2.41	0.000	3.32
Before—3 months post	3.57 ± 2.27	0.000	3.21	3.70 ± 2.75	0.000	2.74
Before—6 months post	4.09 ± 1.90	0.000	4.39	3.61 ± 2.74	0.000	2.69

**Table 3:** Summary of paired samples *t*-tests for self-care scores between groups.

Timepoint comparison of self-care scores	Hydrodissection group (*n* = 23)			Dry-needling group (*n* = 23)		
	M ± SD	*p*-value	Cohen's *d*	M ± SD	*p*-value	Cohen's *d*
Before—1 week post	0.13 ± 0.55	0.266	0.49	0.26 ± 0.62	0.056	0.86
Before—2 weeks post	0.17 ± 0.39	0.043	0.92	0.22 ± 0.60	0.096	0.74
Before—4 weeks post	0.17 ± 0.39	0.043	0.92	0.26 ± 0.62	0.056	0.86
Before—3 months post	0.17 ± 0.39	0.043	0.92	0.26 ± 0.62	0.056	0.86
Before—6 months post	0.17 ± 0.49	0.103	0.72	0.17 ± 0.72	0.257	0.50

### Usual activities

No statistically significant difference was found between the groups on the scores of usual activity. Usual activity scores significantly improved from baseline and at several time points of the two groups. A comparison of each group's respective effect sizes shows that the significant positive changes in the IH + SSE group had the larger effect size across all baseline comparisons. While both groups present significant effects on participants' usual activity, our results suggest that the IH + SSE group has greater clinical significance both in the short and long term (Table 4).

**Table 4:** Summary of paired samples *t*-tests for usual activity scores between groups.

Timepoint comparison of usual activity scores	Hydrodissection group (*n* = 23)			Dry-needling group (*n* = 23)		
	M ± SD	*p*-value	Cohen's *d*	M ± SD	*p*-value	Cohen's *d*
Before—1 week post	0.13 ± 0.81	0.000	2.84	1.04 ± 0.93	0.000	2.30
Before—2 weeks post	1.22 ± 0.74	0.000	3.38	1.04 ± 0.93	0.000	2.30
Before—4 weeks post	0.13 ± 0.76	0.000	3.05	1.13 ± 0.81	0.000	2.84
Before—3 months post	1.17 ± 0.78	0.000	3.09	1.04 ± 0.77	0.000	2.78
Before—6 months post	1.39 ± 0.72	0.000	3.94	0.91 ± 1.00	0.000	1.87

### Pain/discomfort

There was no statistical difference in the scores between the two groups. However, pain/discomfort scores significantly improved within each group compared to baseline levels. The IH + SSE group showed a more significant effect size at two weeks, four weeks, and six months after the intervention, while the DN + SSE group had a higher effect size at three months post-intervention. The IH + SSE group is most clinically effective in improving pain/discomfort scores 2–4 weeks after intervention and in the long-term six months after the intervention (Table 5).

**Table 5:** Summary of paired samples *t*-tests for pain/discomfort scores between groups.

Timepoint comparison of pain/discomfort scores	Hydrodissection group (*n* = 23)			Dry-needling group (*n* = 23)		
	M ± SD	*p*-value	Cohen's *d*	M ± SD	*p*-value	Cohen's *d*
Before—1 week post	0.96 ± 0.77	0.000	2.55	1.26 ± 1.01	0.000	2.55
Before—2 weeks post	1.13 ± 0.76	0.000	3.05	1.30 ± 0.93	0.000	2.88
Before—4 weeks post	1.22 ± 0.90	0.000	2.76	1.26 ± 1.05	0.000	2.45
Before—3 months post	1.17 ± 1.03	0.000	2.33	1.30 ± 1.02	0.000	2.62
Before—6 months post	1.35 ± 0.88	0.000	3.12	1.26 ± 0.86	0.000	2.98

### Anxiety/depression

**Figure 4:**
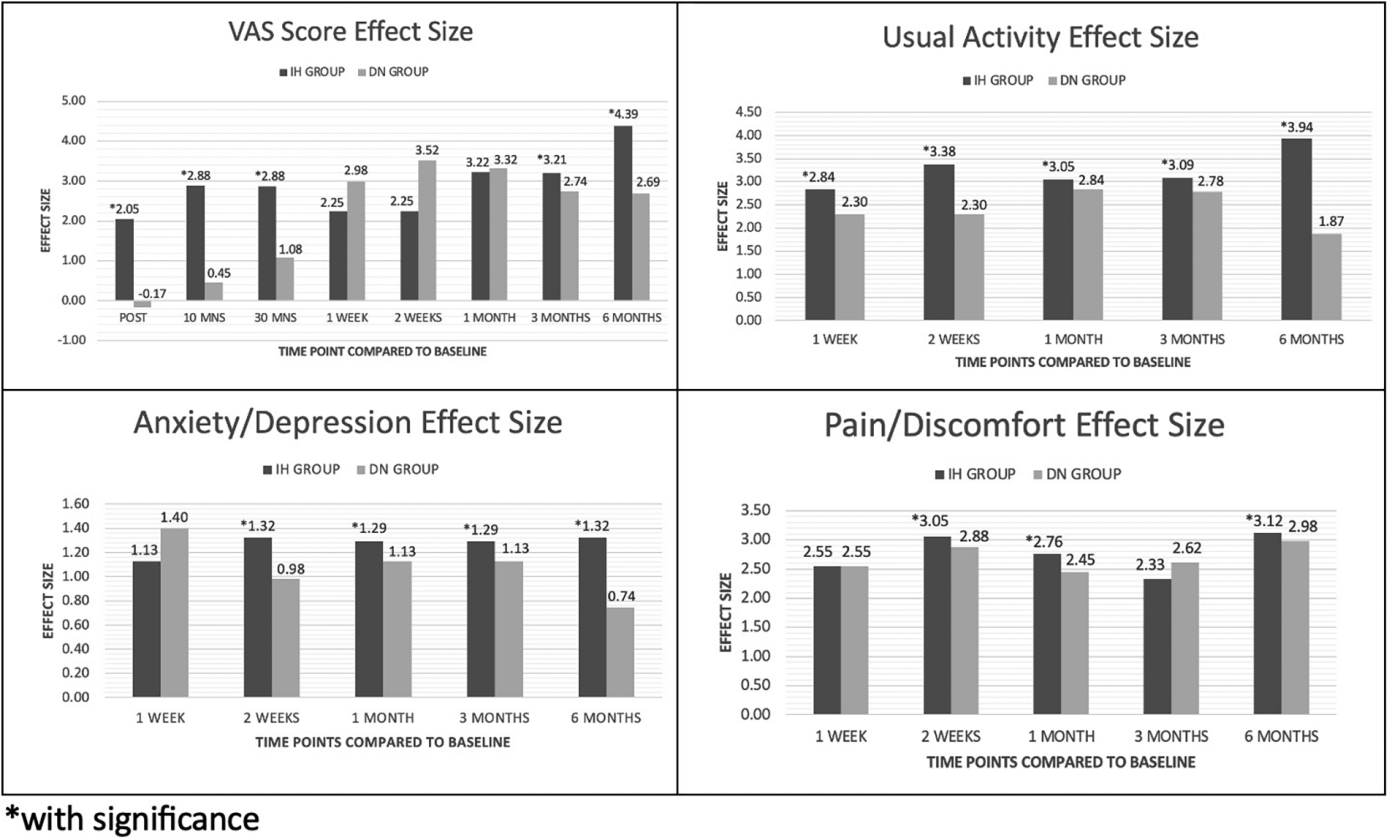
Effect size of hydrodissection and dry needling on VAS, anxiety, usually activity and pain/discomfort.

There was no statistical difference in the scores of the two treatment arms on different time points except for one-week post-intervention, where the IH + SSE group had a higher mean score (1.17 ± 0.39 vs. 1.00 ± 0.00, *p*-value = 0.037). Both groups had a significant mean change from baseline to all time points except for six months post-intervention, where the DN + SSE had no significant change. However, the effect size of the IH + SSE group was shown to be larger at two weeks, four weeks, three months, and six months after the intervention, while the DN + SSE group only had a more significant effect size one week post-intervention (Table 6), (Figure 4).

**Table 6:** Summary of paired samples *t*-tests for anxiety/depression scores between groups.

Timepoint comparison of anxiety/depression scores	Hydrodissection group (*n* = 23)			Dry-needling group (*n* = 23)		
	M ± SD	*p*-value	Cohen's *d*	M ± SD	*p*-value	Cohen's *d*
Before—1 week post	0.52 ± 0.95	0.015	1.13	0.61 ± 0.89	0.003	1.40
Before—2 weeks post	0.61 ± 0.94	0.005	1.32	0.48 ± 0.99	0.031	0.98
Before—4 weeks post	0.57 ± 0.90	0.006	1.29	0.52 ± 0.95	0.015	1.13
Before—3 months post	0.57 ± 0.90	0.006	1.29	0.52 ± 0.95	0.015	1.13
Before—6 months post	0.61 ± 0.94	0.005	1.32	0.39 ± 1.08	0.095	0.74

### Adverse events

Only one patient (4.3%) in the IH + SSE group had neck stiffness in the second-week post-intervention. In the DN + SSE group, one participant (4.3%) felt heaviness on the injection site immediately after the procedure, and one (4.35%) had neck stiffness two months post-procedure. All the adverse events resolved less than a week after doing the exercises. No medications were needed.

## Discussion

Our study showed that both interfascial and dry needling injections effectively decrease pain in the short and long term with significant computed mean change. However, interfascial hydrodissection had a more significant effect size at all times, except for 1, 2, and 4 weeks post-injection, when compared to the control intervention. Furthermore, the pain score was significantly less with interfascial hydrodissection immediately, 10, and 30 min after injection. Of the five dimensions of the EQ-5D-5l, significant mean scores were seen only in self-care for up to six months for the interfascial injection group. Significant mean change was seen in both groups for usual activity, pain/discomfort, and anxiety/ depression, with a higher effect size in the IH + SSE group, compared to the DN + SSE group in almost all time points. To the researchers’ knowledge, this is the first study that determined the difference in the efficacy of dry needling with interfascial injection using lidocaine and saline solution of the upper trapezius in terms of pain, health measures, and adverse events.

Dry needling, a mainstay in treating MPS, effectively decreases pain. Although the mechanism behind trigger point injection is not yet fully understood, it is hypothesized that mechanical and neurophysiological changes occur with its application. Evidence shows that it can lessen the overlapping actin and myosin filaments in the dysfunctional endplates, decreasing persistent muscle contraction. Persistent muscle contraction is associated with the symptom of a taut band (18). Dry needling removes the source of peripheral nociception with a decrease in substances that mediate pain, such as substance *P* and cytokines. This brings about a decrease in dorsal horn activity, which lessens pain perception (18). Two systematic reviews (9, 10) showed that dry needling effectively decreases pain and improves range of motion and quality of life compared to placebo, sham, and no intervention with short-term and medium-term. However, there was no statistical difference between the two groups with the long-term effect, which was from two to six months post-injection.

MPS treatment has recently focused on the trapezius muscle's fascial plane. The muscle fascia coordinates muscular activity as they allow the independent movement of a muscle and form an interfascial space between muscles. It is richly innervated with free and encapsulated nerve endings that can transmit nociceptive signals. Stecco et al. (6) theorized that the layer of loose connective tissue of the fascia has the highest concentration of hyaluronic acid. As a reaction to muscle overuse or injury, large amounts of hyaluronic acid are produced, aggregating into a supermolecular structure and resulting in increased viscosity. This leads to a decrease in the sliding of the fascia, and friction ensues. The friction can irritate the mechanoreceptors and nociceptors found within the fascia (4). Other factors, such as chronic inflammation, peripheral sensitization, muscle hyperexcitability, ischemia, and acidosis, can decrease fascial mobility. It was suggested that decreased fascial mobility is associated with myofascial pain (19). According to Kobayashi et al. (20), the possible mechanism of pain relief in an interfascial block are (1) sodium channel block by a local anesthetic, (2) acid stimuli by injection of a low pH solution, (3) puncture stimuli by needle injection, (4) mechanical stimuli to the myofascial by solution injection, (5) washout of various algesic substances in the interfascial space and (6) separation of the myofascial layers which reduces muscular friction and increases fascial mobility.

Studies on the efficacy of interfascial injection are limited. Two retrospective studies showed that interfascial injection is effective in reducing pain by more than 50% in three months using either 5–10 cc of physiologic saline solution or ten cc of 0.125% bupivacaine (12, 13).

A previous quasi-experimental study by Suarez et al. (14) also demonstrated the technique's efficacy. However, a different solution mixing one cc of 2% lidocaine and five ccs of physiologic saline solution was used with the rationale of having an immediate effect with the use of local anesthetics and a long-term effect with the use of the saline solution by the mechanisms proposed by Kobayashi et al. (20). However, all these three studies had no other intervention group to compare, unlike in the present investigation.

Another key distinguishing characteristic of the present study is our inquiry on the long-term effects of the interfascial injection. A recent investigation by Tantanatip et al. (21) compared physiologic saline interfascial injection with lidocaine trigger point injection in MPS regarding pain using VAS score and neck range of motion. Both interventions significantly decreased VAS scores from 10 min, two weeks, and four weeks after treatment. However, no difference was seen at 2–4 weeks; and no significant difference in the range of motion was observed between the two groups. Our results further support the long-term benefits of interfascial injection in targeting the interfascial space compared to the usual dry needling technique on the trigger nodule. By injecting into the interfascial plane, inflammatory mediators and metabolic by-products that cause pain in the trigger point would be reduced. It can also improve the gliding of the muscles, reducing pain and increasing fascial mobility. We opted to use physiologic saline as the main component of our injectate to reduce adverse reactions from a larger dose of anesthetic agent and reduce the pH level to lessen the pain during injection. One cc of lidocaine was added to the mixture to improve response immediately post-injection.

One possible reason for the long-term effects of interfascial injection is the sustenance of the participants performance of self-stretching exercises, reinforced by the weekly text messages reminding them to perform the exercises. Fernandez de las Peñas and Nijs (18) has recommended that the comprehensive management of MPS should include self-management and exercise programs. Dry needling and interfascial injection improve pain and function in the short term, but comprehensive pain management potentiates the long-term effects with medium to larger effect sizes.

Aside from the VAS score, the researchers were able to assess the effect of treatment on mobility, self-care, usual activity, pain/discomfort, and anxiety/depression, which was quite uncommon among the extant literature. Similar attempts were by Cerezo-Téllez et al. (22), wherein they used the Short Form 36 to assess the health-related quality of life of 128 participants with MPS, comparing dry needling with stretching alone. It showed an improvement in all dimensions of the SF-36 at every point in the dry-needling group. The study of da Costa Santos et al. (23) used the WHOQOL-BREF to assess the quality of life in patients with MPS, either receiving dry needling, ischemic pressure, or the control group. All three groups significantly affected the physiological domain of the WHOQOL-BREF after treatment. Chronic musculoskeletal pain, such as in MPS, can overwhelmingly negatively impact a person's emotional and social well-being. It is suggested that health-related QOL must be an endpoint in clinical trials in treating MPS. Our study extends the current evidence in using interfascial injection as an alternative to dry needling in the treatment of MPS. The findings of this investigation suggest its beneficial effects in reducing pain and improving health outcomes.

The strength of this study is on its ability to determine the effect of both modalities in the short and long term for up to six months. Furthermore, health-related quality of life was also assessed, which gives a broader perspective on how MPS could affect a person's well-being. However, the study did not have a group with self-stretching alone or no intervention at all poses as one of the limitations. Another limitation was that the ultrasound machine available did not have vibration sonoelastography and shear wave elastrography which may provide a deeper understanding of MTrPs. Lastly, the cervical range of motion was not included as an outcome measure. Future investigation is needed in addressing these limitations. In conclusion, interfascial injection and dry needling effectively decrease pain and improve function in patients with MPS. However, interfascial hydrodissection provided a more clinically significant short and long-term pain reduction.

## Data Availability

The raw data supporting the conclusions of this article will be made available by the authors, without undue reservation.
